# Association between smoking and alcohol‐related behaviours: a time–series analysis of population trends in England

**DOI:** 10.1111/add.13887

**Published:** 2017-07-06

**Authors:** Emma Beard, Robert West, Susan Michie, Jamie Brown

**Affiliations:** ^1^ Research Department of Behavioural Science and Health University College London London UK; ^2^ Research Department of Clinical, Educational and Health Psychology University College London London UK

**Keywords:** Alcohol, ARIMAX, ATS, smoking, STS, time series

## Abstract

**Aims:**

This paper estimates how far monthly changes in prevalence of cigarette smoking, motivation to quit and attempts to stop smoking have been associated with changes in prevalence of high‐risk drinking, and motivation and attempts to reduce alcohol consumption in England.

**Design:**

Data were used from the Alcohol and Smoking Toolkit Studies between April 2014 and June 2016. These involve monthly household face‐to‐face surveys of representative samples of ~1700 adults in England.

**Measurements:**

Autoregressive Integrated Moving Average with Exogeneous Input (ARIMAX) modelling was used to assess the association over time between monthly prevalence of (a) smoking and high‐risk drinking; (b) high motivation to quit smoking and high motivation to reduce alcohol consumption; and (c) attempts to quit smoking and attempts to reduce alcohol consumption.

**Findings:**

Mean smoking prevalence over the study period was 18.6% and high‐risk drinking prevalence was 13.0%. A decrease of 1% of the series mean smoking prevalence was associated with a reduction of 0.185% of the mean prevalence of high‐risk drinking 2 months later [95% confidence interval (CI) = 0.033 to 0.337, *P* = 0.017]. A statistically significant association was not found between prevalence of high motivation to quit smoking and high motivation to reduce alcohol consumption (β = 0.324, 95% CI = –0.371 to 1.019, *P* = 0.360) or prevalence of attempts to quit smoking and attempts to reduce alcohol consumption (β = −0.026, 95% CI = –1.348 to 1.296, *P* = 0.969).

**Conclusion:**

Between 2014 and 2016, monthly changes in prevalence of smoking in England were associated positively with prevalence of high‐risk drinking. There was no significant association between motivation to stop and motivation to reduce alcohol consumption, or attempts to quit smoking and attempts to reduce alcohol consumption.

## Introduction

In England in 2015, approximately 17% of the population were smokers and 20% were estimated to drink alcohol at high‐risk levels 1, 2. Smoking and high‐risk alcohol consumption are major causes of a number of fatal diseases including cancer and cardiovascular disease 3. In England, approximately 8000 deaths each year are alcohol‐related and 80 000 per year are attributed to smoking 4, 5. Smoking and high‐risk drinking have been found to be associated at an individual level; this study assessed whether a similar association holds true at a population level over time. Uniquely, England has such data collected monthly and so provides an important context in which to study this.

High‐risk drinkers are substantially more likely to smoke 6, 7, 8, 9, 10 and there is a positive association between the number of cigarettes smoked and alcohol consumption 11, 12. Attempts to quit smoking are less successful among those with alcohol use disorder 13, 14, 15 and episodes of alcohol consumption during a smoking cessation attempt are associated with a greater risk of relapse to smoking 16. Several mechanisms may contribute to the association between alcohol and tobacco use, including genes involved in regulating neurotransmitters, cross‐tolerance and cross‐sensitization to both drugs, conditioning mechanisms in which cravings for alcohol or nicotine are elicited by similar environmental cues, and common psychological and social factors (e.g. personality characteristics and psychiatric conditions) 17, 18, 19, 20, 21, 22. Mechanisms proposed for the negative impact of alcohol on smoking cessation include reduced inhibitory control and an increase in the salience of smoking cues 23, 24.

While the association between high‐risk drinking and smoking has been established at an individual level, much less is known about this association at a population level. This is important, because if there is a causal association in either direction it means that policies that reduce smoking prevalence may have the added benefit of reducing high‐risk drinking or vice versa.

Such a population‐level association may arise from a number of mechanisms. It is common for smokers to be advised to restrict their alcohol consumption when they are attempting to stop smoking 16, 25, 26. Survey data suggest that many smokers follow this advice, with increased rates of attempts to reduce alcohol consumption and less frequent binge drinking among those having recently begun an attempt to quit smoking 27. It is also possible that when smokers stop, this is part of a broader attempt to reduce their health risks that would include a reduction in alcohol consumption as well 28, 29, 30. A third possibility is that not smoking makes it easier to reduce alcohol consumption or vice versa, because each provides a cue for the other or because of pharmacological interactions between nicotine and alcohol 31, 32.

In England, since 2006 population‐level data on smoking prevalence, attempts to stop smoking and motivation to stop smoking have been collected monthly from household surveys. Such a data series is unique, and provides a basis for undertaking fine‐grain time–series analyses on trends in these parameters 33. Since 2014, corresponding data have been gathered on high‐risk drinking and motivation and attempts to reduce alcohol consumption 34. These two data series provide a rare opportunity to examine monthly population‐level associations over time between smoking and high‐risk drinking, and motivations and attempts to change these behaviours.

Although time–series analyses have been used to assess the temporal association between smoking and alcohol consumption at the individual level 35, 36, we are unaware of any previous study which has done so at a population level. The individual‐level studies found significant cross‐correlations between smoking and drinking in which the amount of use of one substance was predicted by use of the other 35. This, and cross‐sectional studies of associations between smoking and alcohol consumption, suggests that we may find positive associations between our smoking and drinking parameters.

If monthly changes in smoking prevalence are associated with changes in prevalence of high‐risk drinking, assessing whether there are corresponding changes in motivation to stop smoking and to reduce alcohol consumption in high‐risk drinkers and attempts to stop smoking and attempts to reduce alcohol consumption in high‐risk drinkers could help to identify the underlying mechanism. Corresponding changes in these variables would suggest that associations between prevalence changes arise from common motivational factors operating at a population level, or reciprocal motivational influences. If there are no corresponding associations between these motivational variables, it suggests that factors relating to capability or the physical or social context may underlie the association. As noted above, this could be because of reductions in smoking and drinking cues or pharmacological interactions.

Thus, this study sought to address the following questions:

1. Is there an association in England between changes in monthly prevalence of smoking and high‐risk drinking?
2. Are there corresponding associations between motivation to stop smoking and motivation to reduce alcohol consumption in high‐risk drinkers, and attempts to stop smoking and attempts to reduce alcohol consumption in high‐risk drinkers?

## Methods

### Design and setting

Data were used from the Smoking and Alcohol Toolkit Studies (STS and ATS) collected between March 2014 and June 2016. The STS and ATS are ongoing studies that involve a series of monthly cross‐sectional household, face‐to‐face, computer‐assisted surveys of representative samples of the population in England aged 16+ 33. Thus, the same participants take part in both surveys. The respondents are recruited using a type of random location sampling, which is a hybrid between random probability and simple quota sampling. England is first split into more than 170 000 ‘Output Areas’, comprising approximately 300 households. These areas are then stratified according to ACORN characteristics and geographical region (http://www.caci.co.uk/acorn/) and are allocated randomly to interviewers. Interviewers visit households within their allocated locality starting at a random point in the area. One member per household is interviewed until interviewers achieve local quotas designed to minimize differences in the probability of participation.

Participants appear to be representative of the population in England, having similar socio‐demographic composition and smoking characteristics to large national surveys based on probability samples, such as the Health Survey for England 33, while drinking characteristics also appear similar at a regional level to other national surveys 37. For further details see: www.smokinginengland.info and www.alcoholinengland.info and the published protocols 33, 34.

### Registration

The analysis plan was pre‐registered on the Open Science Framework (https://osf.io/p5uc8/).

### Participants

Between April 2014 and June 2016, data were collected on 45 414 adults aged 16+ taking part in the STS and ATS who answered questions on their smoking status and alcohol consumption. Fifty‐one per cent [95% confidence interval (CI) = 50.5–51.5] of participants were female, with a mean age of 46.9 (95% CI = 46.7–47.9) years, and 45.5% (95% CI = 45.1–46.0) were in routine or manual jobs or were unemployed. Eight‐seven per cent (95% CI = 86.2–86.8) of participants were of white ethnicity. Of these, 18.6% (95% CI = 18.3–19.0) were current smokers, 20.5% (95% CI = 20.2–20.9) were past year smokers and 13.0% (95% CI = 12.7–13.3) were high‐risk drinkers [defined as having an Alcohol Use Disorders Identification Test (AUDIT) score greater than 8].

### Ethical approval

Ethical approval for the STS was granted originally by the UCL Ethics Committee (ID 0498/001). Approval for the ATS was granted by the same committee as an extension of the STS. The data are not collected by UCL and are anonymized when received by UCL. Explicit verbal agreement and willingness to answer questions voluntarily is recorded electronically by Ipsos Mori, the company administering the survey. This standard protocol was agreed by the UCL ethics committee. Participants are also given a printed information sheet.

### Measures

Participants were asked whether they smoked or had smoked cigarettes (including hand‐rolled) daily or non‐daily during the past year and to complete the AUDIT 38. The AUDIT identifies people who could be classed as dependent, harmful or hazardous drinkers and has demonstrated validity, high internal consistency and good test–retest reliability across gender, age and cultures 39, 40, 41, 42. Those scoring 8 or more were classed as high‐risk drinkers. This is the conventional cut‐off threshold 40, 43, 44, 45. The prevalence of smoking and high‐risk drinking in each month was obtained by counting the number of respondents who reported smoking and an AUDIT score greater than or equal to 8, respectively.

Past year smokers were asked: ‘How many serious attempts to stop smoking have you made in the last 12 months? By serious attempt I mean you decided that you would try to make sure you never smoked again. Please include any attempt that you are currently making and please include any successful attempt made within the last year’. The prevalence of quit attempts was obtained by counting the number of respondents who reported having made one or more quit attempt in the past 12 months.

Current smokers completed the single item Motivation to Stop Scale (MTSS), which has been shown to have good predictive validity for quit attempts 46. It asks: ‘Which of the following best describes you? I REALLY want to stop smoking and intend to in the next month; I REALLY want to stop smoking and intend to in the next 3 months; I want to stop smoking and hope to soon; I REALLY want to stop smoking but I don't know when I will; I want to stop smoking but haven't thought about when; I think I should stop smoking but don't really want to; I don't want to stop smoking; don't know’. The prevalence of high‐motivation to quit in each month was obtained by counting the number of respondents who reported that they intended to stop smoking within 3 months.

Between April 2014 and May 2014 high‐risk drinkers were asked: ‘How many serious attempts to cut down on your drinking alcohol have you made in the last 12 months? By serious attempt I mean you decided that you would try to make sure you reduced the amount you drank permanently. Please include any attempt that you are currently making and please include any successful attempt made within the last 12 months’. In subsequent waves participants were asked: ‘How many attempts to restrict your alcohol consumption have you made in the last 12 months (e.g. by drinking less, choosing lower‐strength alcohol or using smaller glasses)? Please include all attempts you have made in the last 12 months, whether or not they were successful, AND any attempt that you are currently making’. They were also asked: ‘During your most recent serious attempt to restrict your alcohol consumption, was it a serious attempt to cut down on your drinking permanently?’. The prevalence of attempts to reduce alcohol consumption was obtained by counting the number of respondents who reported having made one or more attempt to reduce in the past 12 months.

High‐risk drinkers were also asked: ‘Which of the following best describes you? I REALLY want to cut down on drinking alcohol and intend to in the next month; I REALLY want to cut down on drinking alcohol and intend to in the next 3 months; I want to cut down on drinking alcohol and hope to soon; I REALLY want to cut down on drinking alcohol but I don't know when I will; I want to cut down on drinking alcohol but haven't thought about when; I think I should cut down on drinking alcohol but don't really want to; I don't want to cut down on drinking alcohol’. The prevalence of high‐motivation to reduce in each month was obtained by counting the number of respondents who reported that they intended to reduce their consumption within 3 months.

### Analyses

If there were data missing on either smoking or drinking variables, the case was classified as missing in calculating the prevalence figures. The missing value rate was 0.05% (*n* = 22) for smoking and 0.63% (*n* = 285) for high‐risk drinking, 0% (*n* = 0) for motivation to stop smoking or reduce alcohol consumption, and 2.95% (*n* = 277) for attempts to stop smoking and 0% (*n* = 0) for attempts to reduce alcohol consumption. All data were analysed in R version 3.3.1 using Autoregressive Integrated Moving Average with Exogeneous Input (ARIMAX) modelling to assess (1) the association between prevalence of smoking and high‐risk drinking, (2) prevalence of high motivation to quit and to reduce alcohol consumption and (3) prevalence of attempts to quit smoking and prevalence of attempts to reduce alcohol consumption. ARIMAX is an extension of Autoregressive Integrated Moving Average Analysis (ARIMA), which produces forecasts based upon prior values in the time–series (autoregressive terms; AR) and the errors made by previous predictions (moving average terms; MA). We followed a standard ARIMAX modelling approach 47.

The series were first log‐transformed to stabilize the variance and, if required, ‘first differenced’ and ‘seasonally differenced’. First differencing involves calculating the change between one observation and the next, while seasonal differencing involves calculating the change between 1 year and the next. Outliers were then identified and removed from the analysis. This is best practice in time–series analyses and was part of the pre‐planned analysis plan 48, 49. Outliers were identified using (1) the ‘tsoutliers’ package 50, which implements an iterative procedure of anomaly identification and model estimation based on the approach described in Chen & Liu 51 and (2) box‐plots. March 2014 (wave 1) was identified as an outlier. Given that the data are based on surveys that are subject to sampling variation and it is unlikely that there would be very large changes in smoking prevalence or high‐risk drinking in a single month, this outlier probably reflects sampling error. Sensitivity analyses were also run, which included the outlier, and are given in the footnote to Table 2. The autocorrelation and partial autocorrelation functions were examined to determine the seasonal and non‐seasonal MA and AR terms. To identify the most appropriate transfer function for the continuous explanatory variables the sample cross‐correlation function was checked and models with varying lags compared using the Akaike Information Criterion (AIC). The assumption of weak exogeneity, assessed using the Granger Causality Test, was met, i.e. the independent or input time–series did not display evidence of receiving feedback from the dependent or output time–series. In other words, prevalence of high risk‐drinking, motivation to reduce alcohol consumption and attempts to reduce alcohol consumption were not predictive of the corresponding smoking variables. As both the input and output time–series were log‐transformed, the coefficients can be interpreted as estimates of the percentage change from the mean of the series in the outcome of interest for every percentage change in the input series (i.e. in terms of elasticity).

### Power/sample size

There are no established recommendations as to the number of data points required for ARIMAX analysis, but for similar ARIMA models it has been argued that the approach is suitable for short time–series providing there are more observation periods than parameters 52. The current data were aggregated monthly, and therefore even two AR and two MA terms for the model and seasonality components would still have provided sufficient power assuming no lags.

## Results

**Figure 1:**
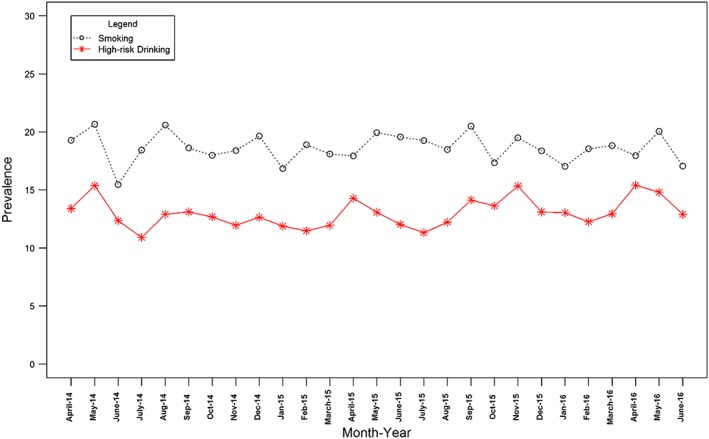
Prevalence over time of smoking and high‐risk drinking. [Colour figure can be viewed at wileyonlinelibrary.com]

**Figure 2:**
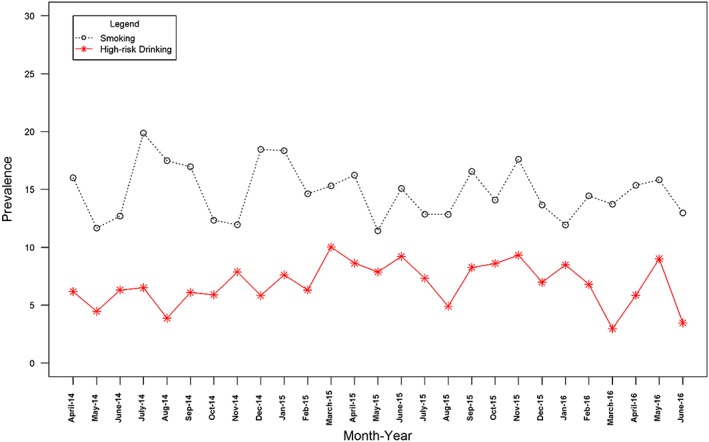
Prevalence over time of high motivation to quit smoking and to reduce alcohol consumption. [Colour figure can be viewed at wileyonlinelibrary.com]

**Figure 3:**
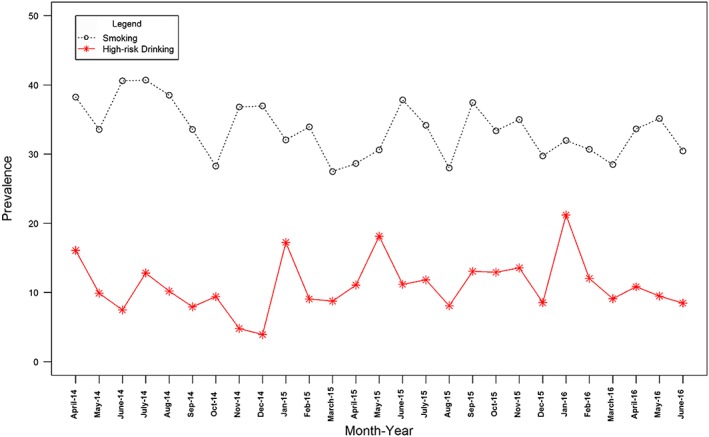
Prevalence over time of attempts to quit smoking and attempts to reduce alcohol consumption. [Colour figure can be viewed at wileyonlinelibrary.com]

Figures 1, 2, 3 show the changes in monthly prevalence of (1) smoking and high‐risk drinking, (2) high motivation to quit smoking and high motivation to reduce alcohol consumption and (3) attempts to stop smoking and attempts to reduce alcohol consumption, while Table 1 shows the mean, end‐ and start‐points for these series.

**Table 1:** Prevalance of smoking,high‐risk drinking, attempts to stop smoking, attempts to reduce alcohol consumption, motivation to quit smoking and motivation to reduce alcohol consumption during the study period.

Treatment	April 2014%	June 2016%	Mean % (SD)
Smoking	19.30	17.10	18.60 (1.25)
High‐risk drinking	13.40	12.90	13.00 (1.23)
High motivation to quit smoking	16.00	13.00	14.90 (2.34)
High motivation to reduce alcohol consumption	6.20	3.50	6.80 (1.86)
Attempts to quit smoking	38.20	30.40	33.60 (3.92)
Attempts to reduce alcohol consumption	16.10	8.50	11.00 (3.87)

SD = standard deviation.

There was a significant association between smoking and high‐risk drinking prevalence: every 1% decrease from the series mean smoking prevalence was associated with a 0.19% decrease from the mean in prevalence of high‐risk drinking 2 months later (see Table 2). Converting this to a percentage point change, a decline in the prevalence of smoking of 1 percentage point (from 18.6 to 17.6%) was associated with a 0.1 percentage point decrease in the prevalence of high‐risk drinking. This is calculated as follows: a 1 percentage point prevalence reduction in smoking prevalence is equivalent to a 5.38% decline from the series mean (i.e*.* $\frac{17.7-18.6}{18.6}$ × 100), which is associated with a 1.02% (i.e. 0.19 × 5.38) decline from the series mean of high‐risk drinking prevalence. The series mean was 13.0%, of which 1.02% is 0.1%.

We did not find statistically significant associations between motivation to stop smoking and motivation to reduce alcohol consumption, or between attempts to stop smoking and attempts to reduce alcohol consumption (see Table 2).

**Table 2:** Estimated percentage point changes in the proportion of high‐risk drinkers, high‐risk drinkers with high motivation to reduce alcohol consumption and high‐risk drinkers having made an attempt to reduce their consumption from April 2014 until the June 2016 as a function of smoking parameters, based on Autoregressive Integrated Moving Average with Exogeneous Input (ARIMAX) models.

	Smoking^a^	High motivation to quit smoking	Attempts to stop smoking
	Percentage change per 1% change in the exposure (95% CI) P	Percentage change per 1% change in the exposure (95% CI) P	Percentage change per 1% change in the exposure (95% CI) P
High‐risk drinking (lag of 2) High motivation to reduce alcohol consumption (no lag) Attempts to reduce alcohol consumption (no lag)	0.185 (0.033 to 0.337) 0.017	0.324 (−0.371 to 1.019) 0.360	−0.026 (−1.348 to 1.296) 0.969
Best‐fitting model	ARIMAX (0,1,0)(0,0,0)^12^	ARIMAX (0,1,1)(0,0,0)^12^	ARIMAX (0,1,0)(0,0,0)^12^
Non‐seasonal (*P*) AR MA Seasonal (*P*) AR MA	NA NA NA NA	NA < 0.001 NA NA	NA NA NA NA

95% CI = 95% confidence interval; MA = moving average; AR = autoregressive; an AR(1) means that the value of a series at one point in time is the sum of a fraction of the value of the series at the immediately preceding point in time and an error component, while MA(1) means that the value of a series at one point in time is a function of a fraction of the error component of the series at the immediately preceding point in time and an error component at the current point in time.

a Similar results were found with a lag of 1 and 0 (β 0.384, 95% CI = 0.054 to 0.713, *P* = 0.022 versus β 0.368, 95% CI = 0.016 to 0.719, *P* = 0.041); effect sizes were also similar and in the same direction when the outlier (March 2014) was included in the analysis: (1) smoking and high‐risk drinking prevalence: 0.322, 95% CI = –0.019 to 0.641, *P* = 0.064; (2) motivation to quit smoking and motivation to reduce alcohol consumption: 0.374, −0.321 to 1.069, *P* = 0.291; (3) attempts to stop smoking and attempts to reduce alcohol consumption: −0.026, −1.323 to 1.271, *P* = 0.969. NA = not applicable.

## Discussion

The change in prevalence of smoking was associated positively with prevalence of high‐risk drinking during a monthly time–series between 2014 and 2016 in England. Statistically significant associations were not found between changes in motivation to stop smoking and to reduce alcohol consumption in high‐risk drinkers, or between attempts to stop smoking and attempts to reduce alcohol consumption.

Although we cannot infer cause and effect, ARIMAX modelling affords the ability to model the temporal ordering between changes in variables being studied 47. In this study, prevalence of smoking during the last 2 months was found to predict the current prevalence of high‐risk drinking. This suggests that if there is a causal association it is from smoking prevalence to high‐risk drinking, rather than vice versa. However, it is also possible that unmeasured variables account for the change in both smoking and high‐risk drinking. Price increases applying at the same time to tobacco and alcohol may be one factor 53, 54. However, the price of cigarettes has increased linearly over time, so its impact would have been removed by differencing the time–series (that is, using the difference between successive values of the outcome variables rather than the values themselves) 34. Several alcohol policies came into effect during the study period, including the removal of financial incentives aimed at encouraging general practitioners to screen their patients for heavy drinking, a ban on the sale of alcohol below the total cost of duty and value‐added tax (VAT) combined in May 2014, and strengthening of local alcohol licensing policies 55, 56, 57, 58. Tobacco policies include the Children and Families Act which, in 2015, made it an offence for an adult to buy tobacco for anyone aged under 18 years (including proxy purchasing) and the revised Tobacco Products Directive in 2016 which mandated the introduction of plain packaging, a ban on packs containing fewer than 20 cigarettes and stricter e‐cigarette regulation 59, 60. To our knowledge, no policies were introduced that would have been expected to influence directly both alcohol consumption and smoking, and it is not clear that cultural shifts would have occurred that could explain the monthly changes. Seasonal influences were removed, so motivation to reduce unhealthy behaviours around the New Year would not explain the findings.

The failure to find a clear association between attempts to stop smoking and attempts to reduce alcohol consumption goes against individual‐level findings in the literature 13, 14, 15. This may be because of low statistical power, with the samples in this analysis limited to smokers and high‐risk drinkers which comprises less than one‐fifth of the sample. It may also be due to limits in the precision of the measures. Although the validity of the MTSS has been demonstrated 46, studies have found large individual level variations in motivation and intention to change behaviour and this may also translate to a population level 61, 62. It will be important to monitor these trends closely over time as more data are collected. With regard to self‐reported attempts to stop smoking, there is evidence that these are forgotten relatively rapidly if they fail 63, which would reduce the ability to pick up associations.

The main policy implication of the findings is that achieving reductions in smoking prevalence may yield health benefits that go beyond smoking. There has been discussion in the literature about whether smoking and alcohol use are complementary or substitutable 64, 65. If they are substitutes, then a policy measure aimed at reducing smoking could lead to increased alcohol consumption. If they are complementary, however, a smoking ban could have the desirable effect of also reducing the consumption of alcohol. Our finding of a positive association between prevalence of smoking and high‐risk drinking provides some support for a complementary effect 65, 66. If the findings are confirmed and are found to reflect a causal association, models of return on investment from smoking cessation policies would need to take this into account. Thus, in future, it would be of interest to assess the impact of population‐level policies targeting either behaviour on the prevalence of the other in an interrupted time–series design.

This study had several limitations. First, it did not adjust for population‐level policies. There was only a comparatively short time–series available and a danger of over‐parameterizing the model. Secondly, the STS and ATS require participants to recall past alcohol and smoking behaviour, which may introduce bias. Thirdly, the findings may not generalize to other countries. England has a strong tobacco control climate and generally high motivation to quit among smokers. In countries with weaker tobacco control, different effects may be observed.

## Conclusions

Changes in prevalence of smoking in England from 2014 to 2016 across a monthly time–series were associated positively with changes in prevalence of high‐risk drinking. The temporal ordering of the association suggests that if there is a causal association it is in the direction of changes in smoking prevalence driving changes in alcohol use. No clear association was found between motivation to stop smoking and motivation to reduce alcohol consumption in high‐risk drinkers or attempts to stop smoking and attempts to reduce alcohol intake in high‐risk drinkers. This suggests that the association between changes in prevalence of smoking and high‐risk drinking may reflect capability and environmental factors rather than motivation to attempt a change in behaviour.

### Declaration of interests

R.W. undertakes consultancy and research for and receives travel funds and hospitality from manufacturers of smoking cessation medications (Pfizer, GSK and J&J); E.B. and J.B. have received unrestricted research funding from Pfizer. R.W.'s salary is funded by Cancer Research UK. S.M.'s salary is funded by Cancer Research UK and by the National Institute for Health Research (NIHR)'s School for Public Health Research (SPHR). B. and J.B. are funded by CRUK (C1417/A14135). E.B. is also funded by NIHR's SPHR and J.B. by the Society for the Study of Addiction. All authors declare there are no other relationships or activities that could appear to have influenced the submitted work.

### Data sharing

For access to the Smoking Toolkit Study (STS) or the Alcohol Toolkit Study (ATS) please contact the lead author or Professor Robert West robertwest100@googlemail.com
